# Presumed Acute Coronary Syndrome in a Patient With Bilateral Pulmonary Embolism

**DOI:** 10.7759/cureus.108657

**Published:** 2026-05-11

**Authors:** Igal Gorbut, Esdras Panchame Pavon, Michael Tedla, Muhammad Faiz, Luke Ong

**Affiliations:** 1 Internal Medicine, Saba University School of Medicine, The Bottom, BES; 2 Internal Medicine, HCA Florida Blake Hospital, Bradenton, USA; 3 Cardiology, HCA Florida Blake Hospital, Bradenton, USA

**Keywords:** acute pulmonary embolism, coronary catheterization, cta chest, ecg abnormalities, ekg abnormalities, non-st elevated myocardial infarction

## Abstract

Background: Acute pulmonary embolism (PE) represents a significant cause of cardiovascular mortality, requiring rapid intervention often preceding definitive diagnosis. While traditional risk factors, including immobilization, malignancy, and thrombophilia, are well-established, PE rarely presents as a diagnostic mimic of acute coronary syndrome (ACS).

Methods: We present a case of a 59-year-old male patient with symptoms and electrocardiogram (ECG) findings highly suggestive of ACS. Emergency left heart catheterization revealed no obstructive coronary artery disease (CAD). Subsequent computed tomography (CT) angiography of the chest identified extensive bilateral pulmonary emboli involving the segmental arteries. Retrospective history revealed recent prolonged immobilization as the primary provocative factor.

Results: The patient was stabilized and transitioned to oral anticoagulation (apixaban). He was discharged in stable condition for outpatient cardiovascular follow-up.

Conclusion: PE-induced right ventricular strain can manifest with ECG changes that closely simulate ACS, posing a significant diagnostic challenge. Physicians should consider PE in patients presenting with atypical ACS features. Standardizing management protocols for PE-associated cardiac strain should be considered to prevent diagnostic delay and optimize clinical outcomes.

## Introduction

Acute pulmonary embolisms (PEs) are a common reason for hospitalization and may be potentially fatal. When suspecting a PE, it is critical to stabilize the patient and begin anticoagulation even prior to the confirmation of the diagnosis. PE falls under multiple categories and risk stratification, mainly high, moderate, or low risk. Hemodynamically unstable PE encompasses a combination of systolic blood pressure of <90 mmHg for >15 minutes or a drop of >40 mmHg from baseline. Patients with high-risk PEs are extremely unstable and may progress to require mechanical ventilation or suffer a cardiac arrest. Low-risk PEs do not meet the definition of hemodynamic instability and normally present with small embolic obstructions, stable blood pressure, normal right ventricular size and function, and normal cardiac biomarkers. Moderate-risk PEs, such as in our patient, have extensive emboli, tachycardia, right ventricular dysfunction, and abnormal biomarkers with borderline blood pressure. Strain on the right side of the heart is a source of ischemia that leads to EKG changes that classically appear as S wave changes in the first limb lead and Q and T-waved changes in the third limb lead [1]. 

Risk factors that lead to both deep vein thrombosis (DVT) and PE are injury to a vein caused by fractures, muscle injury, or major surgery, especially involving the abdomen, pelvis, hips, or legs. The resulting limited blood flow caused by decreased mobility, prolonged sitting, and paralysis especially exacerbates the risk. Medications such as estrogen-containing contraceptives and hormone replacement therapy increase the risk of hypercoagulability. Pregnancy is another risk factor, with the risk even being prominent up to three months after giving birth. Chronic medical conditions such as heart disease, lung disease, cancer, and inflammatory bowel disease are another source of increased risk of hypercoagulability. Other factors to consider are a previous history of both PE and DVTs, inherited clotting disorder, age, obesity, and an indwelling catheter, especially in a central vein [2].

The incidence of concurrent acute coronary syndrome (ACS), which encompasses both ST-elevated myocardial infarction (STEMI) and non-ST-elevated myocardial infarction (NSTEMI), and PE is extremely rare. Out of 2.5 million hospitalizations for ACS, approximately 1% were diagnosed with venous thromboembolism (VTE) [3]. Goal-directed therapy is not well-established for the management of these conditions simultaneously, and the approach is often on a case-by-case basis. We are presenting a case of a patient with an initial presentation of elevated troponin with EKG changes caused by an underlying bilateral PE.

## Case presentation

**Figure 1:**
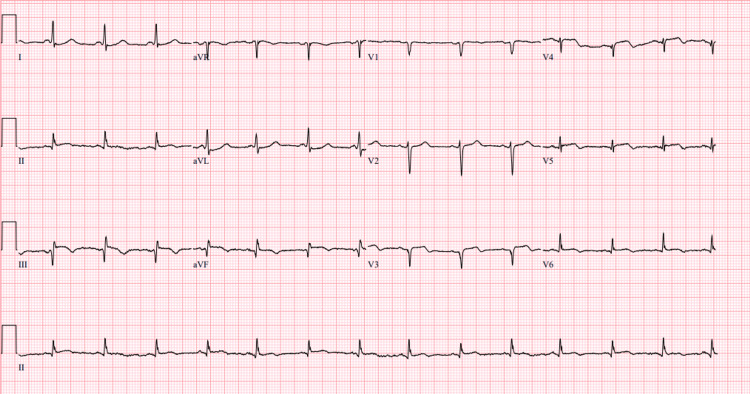
Admission ECG ECG: electrocardiogram A 12-lead ECG demonstrating an inferior and precordial ST-segment elevations in leads II, III, aVF, and V3-V4. Reciprocal ST-depression noted in lead aVL. Diagnostic markers for the right ventricular strain are present, including the S1Q3T3 sign (prominent S-wave in lead I; Q-wave and T-wave inversion in lead III) and widespread T-wave inversions in the inferolateral leads (V3-V6 and lead III)

A 59-year-old male patient with no significant past medical history or documented home medications presented to the emergency department with a one-week history of progressive dyspnea on exertion. The patient reported a diagnosis of influenza seven days prior to the onset of current symptoms. Upon initial evaluation, the patient was hemodynamically stable: blood pressure was 113/78 mmHg, heart rate was 83 beats per minute, and peripheral oxygen saturation (SpO2) was 93% on room air. Laboratory investigations revealed a significantly elevated cardiac troponin level of 2600 ng/L. The electrocardiogram (ECG) demonstrated ST-segment deviations in leads II, III, aVF, and V3-V4 (Figure 1).

**Figure 2:**
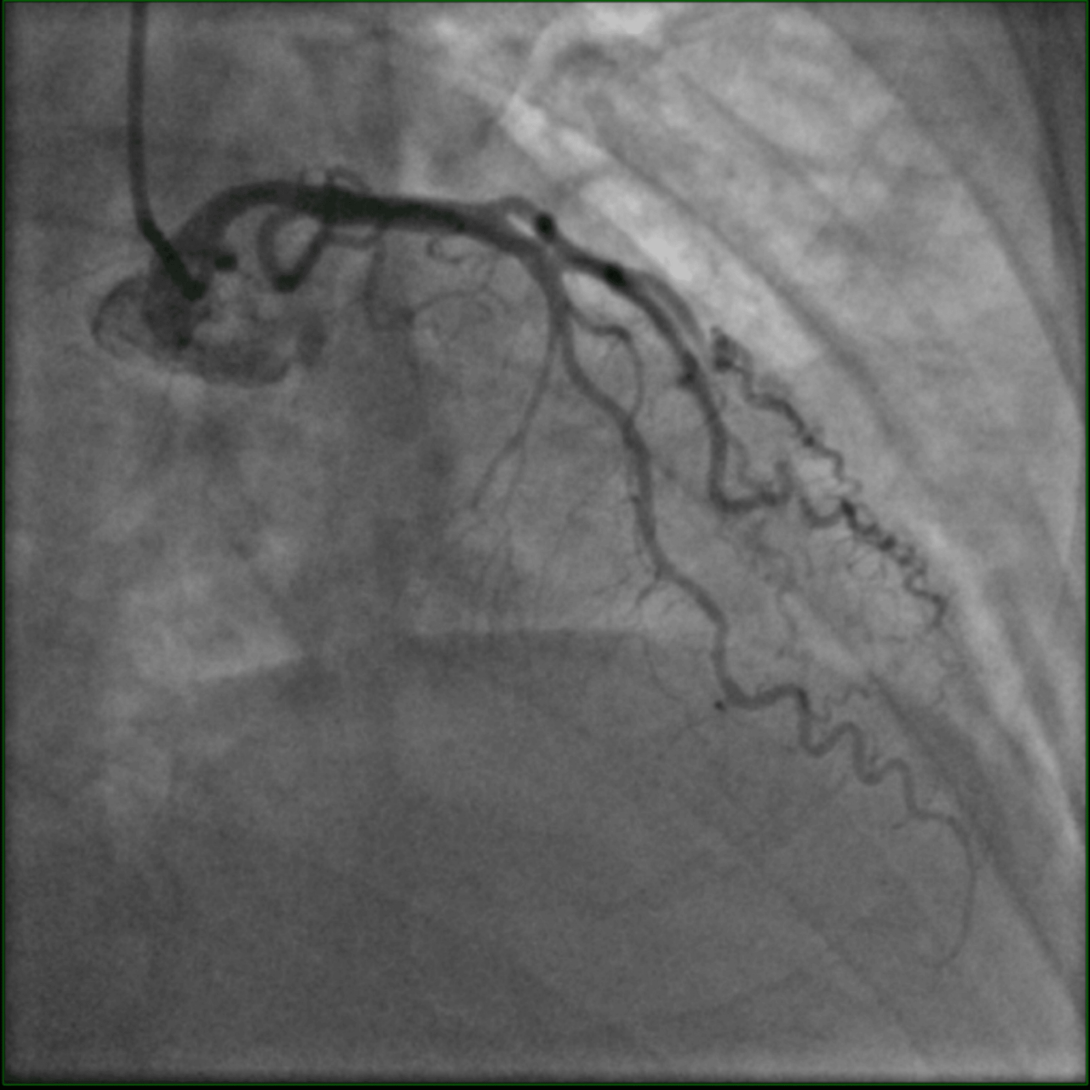
Invasive coronary angiography Selective angiography of the left coronary system demonstrating no-obstruction in the left main artery bifurcation into the left anterior descending and left circumflex arteries. No hemodynamically significant stenosis or acute plaque rupture is visualized

Based on the clinical presentation and ECG findings, a presumptive diagnosis of ACS was made. An STEMI protocol was activated, and the patient was immediately started on intravenous heparin and aspirin. Following cardiology consultation, the ECG was interpreted as concerning for an NSTEMI; however, given the clinical acuity, the patient was taken emergently for coronary angiography and left heart catheterization. Left heart catheterization demonstrated nonobstructive coronary artery disease (CAD), with less than 10% stenosis in all major epicardial vessels. Left ventriculography revealed a preserved left ventricular ejection fraction (LVEF) of 55% and a left ventricular end-diastolic pressure (LVEDP) of 13 mmHg (Figure 2). Coronary angiography excluded obstructive CAD as the underlying etiology of the patient's presentation. The absence of hemodynamically significant stenosis or evidence of acute plaque rupture confirmed that the initial clinical suspicion of a primary ACS was inaccurate.

**Figure 3:**
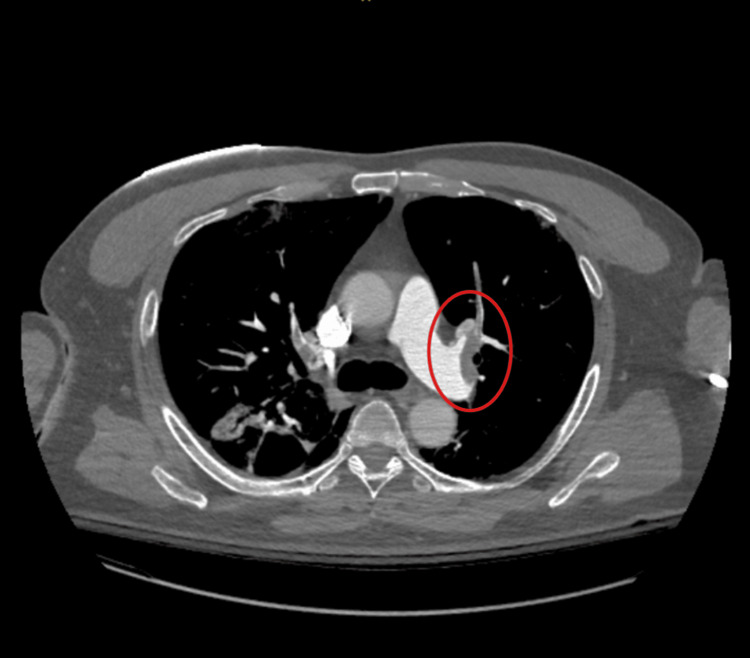
Computer tomography with angiography of the chest and pulmonary vasculature, axial view The image reveals acute pulmonary embolism, highlighted by a red circle, revealing a significant filling defect located at the bifurcation of the left main pulmonary artery. Thromboembolism extends distally, involving the left upper lobe segmental arteries

**Figure 4:**
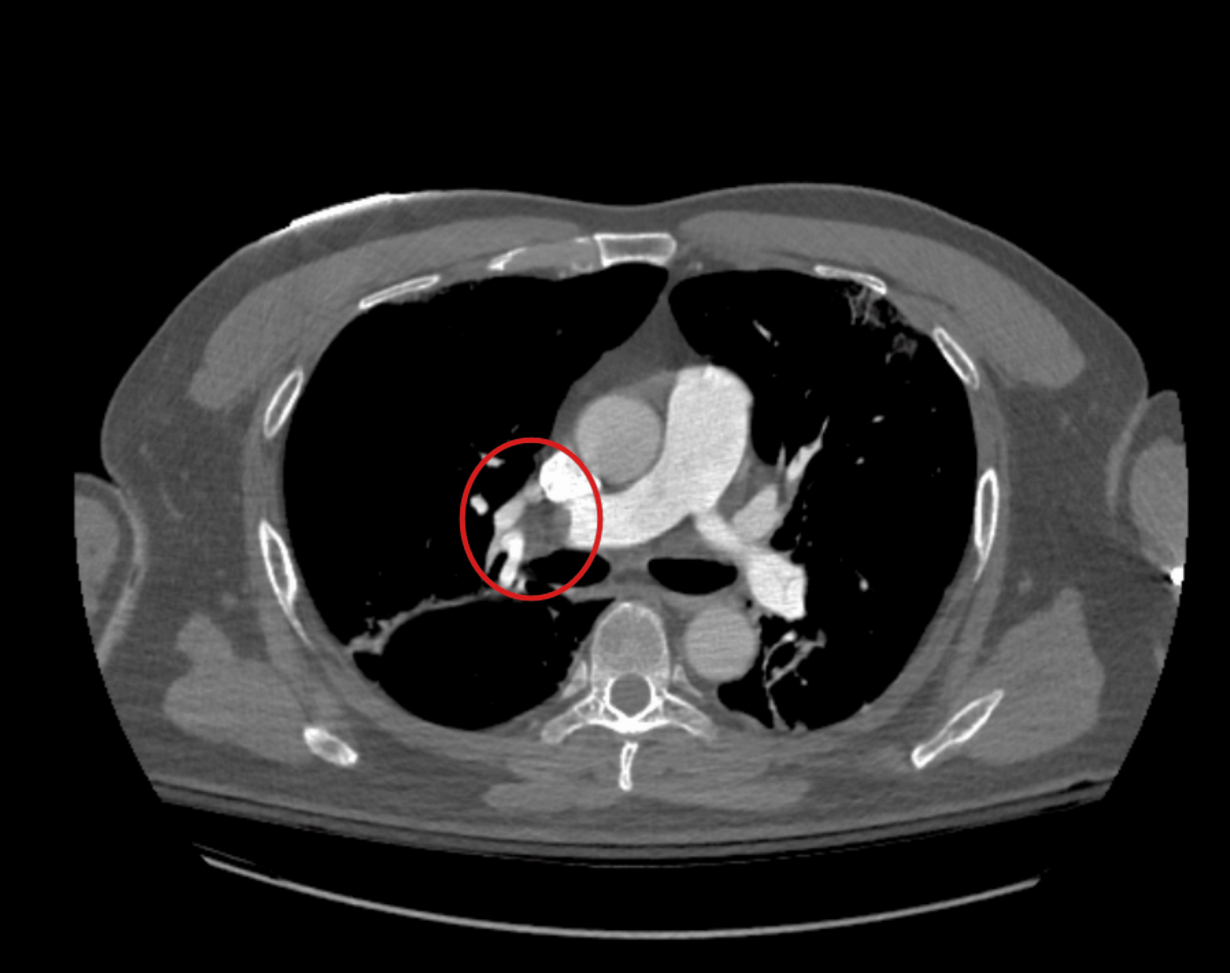
Computer tomography with angiography of the chest and pulmonary vasculature Image reveals acute pulmonary embolism. A red circle highlights a significant filling defect located at the bifurcation of the right main pulmonary artery, extending into both upper and lower lobe segmental arteries

Given the discordant findings from the initial suspicion, the differential diagnosis was expanded to include myocardial Infarction with nonobstructive coronary arteries (MINOCA), viral myocarditis (given the patient's preceding viral infection), and acute PE. Subsequent imaging with CT angiography of the chest revealed bilateral PE's originating at the bifurcation of the main pulmonary arteries. The emboli extended distally into the left upper lobe segmental arteries (Figure 3) as well as the right upper lobe and right lower lobe segmental arteries (Figure 4). These findings suggest that the myocardial injury, initially manifesting as troponin elevation and EKG changes, was a secondary phenomenon attributable to the acute PE mimicking ACS rather than an intrinsic coronary artery event.

Upon further questioning to identify potential provoked risk factors for venous thromboembolism (VTE), the patient reported a recent 11-hour prolonged automobile journey shortly before the onset of symptoms. Notably, CT imaging showed no evidence of pulmonary malignancy or intrathoracic lymphadenopathy. The patient’s age-appropriate health screenings were up to date, including a normal colonoscopy performed two years prior to the current admission. Laboratory risk stratification revealed a thyroid-stimulating hormone (TSH) level of 1.18 uIU/L and elevated triglycerides of 126 mg/dL. A hemoglobin A1c of 7.9% confirmed a new diagnosis of type 2 diabetes mellitus (Table 1).

**Table 1:** Summary of laboratory parameters, metabolic profile, and hemodynamic data during hospitalization H: high; N: within normal range; L: low; LDL: low-density lipoprotein; HDL: high-density lipoprotein; LVEDP: left ventricular end-diastolic pressure

Parameter	Patient value	Reference range
Troponin I high sensitivity	2617 ng/L (H)	<54 ng/L
Thyroid-stimulating hormone	1.18 uIU/mL (N)	0.36-3.74 uIU/mL
Triglycerides	126 mg/dL (N)	30-150 mg/dL
LDL cholesterol	46 mg/dL (N)	0-99 mg/dL
HDL cholesterol	30 mg/dL (L)	40-59 mg/dL
Hemoglobin A1c	7.9% (H)	4.5-6.2%
LVEDP	13 mmHg (H)	3-12 mmHg

The patient was classified as having a submassive PE based on the presence of elevated troponins and the absence of hemodynamic instability or other signs of end-organ damage. Following a multidisciplinary discussion, a shared decision-making approach was employed to determine the optimal management strategy. Despite the submassive nature of the PE, the patient expressed a strong preference for a less invasive management plan. This preference was rooted in his high baseline functional status, regular physical activity, and the absence of pre-existing comorbidities prior to hospitalization. Given his sustained hemodynamic stability and the resolution of presenting symptoms, the clinical team concurred that a conservative approach with anticoagulation is appropriate, avoiding the inherent risks associated with catheter-directed therapy or systemic thrombolysis. While mechanical thrombectomy or catheter-directed therapies are viable options, the inherent risks of cardiac chamber or valvular injury during manipulation, major hemorrhage, and iatrogenic pulmonary trauma were considered. This decision was supported by the rapid resolution of acute symptoms and consideration of avoiding potential permanent morbidity or mortality associated with the invasive maneuvers, which the patient opposed. 

To identify the primary source of the emboli, a lower extremity venous duplex ultrasound was performed. The study revealed an occlusive deep vein thrombosis (DVT) within the left popliteal vein; the right lower extremity was unremarkable. Subsequently, the patient was transitioned from a continuous heparin infusion to oral apixaban, initially 10mg twice daily for seven days, followed by 5 mg twice daily, for long-term anticoagulation.

CT scan of the chest also demonstrated patchy bibasilar airspace opacities and focal peripheral opacities within the superior lobes bilaterally. Given the patient’s initial respiratory distress and these radiographic findings, a presumptive diagnosis of community-acquired pneumonia (CAP) was made. He was started on an empiric intravenous antibiotic regimen consisting of ceftriaxone and azithromycin during his hospitalization. This infectious process was considered a significant contributing factor to the patient’s initial presentation. Upon clinical stabilization and the resolution of acute symptoms, the patient was transitioned to oral cefdinir to be completed as an outpatient. 

Comprehensive counseling was provided regarding the necessity of close follow-up with his primary care physician. Although the patient’s health screenings were largely up to date, a repeat outpatient colonoscopy was recommended to further evaluate for occult gastrointestinal malignancy as a potential contributing factor to his hypercoagulable state.

The postdischarge management plan included a follow-up with a cardiologist for an outpatient transthoracic echocardiogram (TTE). Although indicated during the hospitalization, the echocardiogram was deferred at the patient's request to accommodate urgent professional and personal obligations. The follow-up imaging remains a clinical priority to establish a formal baseline diagnosis for cardiac function aside from what was obtained during the left heart catheterization. Given the initial presentation of a submassive PE with myocardial strain, serial TTE is essential to monitor for potential long-term sequelae, specifically the development of chronic pulmonary hypertension (CTEPH) or persistent ventricular strain. 

## Discussion

PE mimicking ACS, encompassing both STEMI and NSTEMI, is a rare clinical phenomenon that presents significant therapeutic challenges. In the absence of standardized guidelines, clinicians often prioritize the management of acute PE over the simultaneous treatment of both conditions. While high-risk PE (defined by hemodynamic instability) necessitates urgent systemic thrombolysis or catheter-directed thrombectomy, intermediate-risk cases are typically managed with anticoagulation and vigilant observation. Current guidelines advocate for low-molecular-weight heparin or direct oral anticoagulants (DOACs) as first-line therapy for hemodynamically stable patients [1]. However, specific evidence-based protocols for PE-induced myocardial injury remain undefined, requiring clinicians to rely on individual judgment. In this case, the patient was managed according to intermediate-risk PE protocols using DOAC therapy.

Surgical embolectomy is indicated for patients with PE when thrombolytic therapy is contraindicated or when clinical deterioration occurs despite anticoagulation. Interventional approaches are broadly categorized into catheter-directed therapies and open surgical embolectomy. Catheter-directed techniques involve thrombus fragmentation and aspiration through large-lumen catheters. In contrast, surgical embolectomy is reserved for patients who have failed or have contraindications to both systemic and catheter-directed lysis. Anatomically, surgical intervention is most effective for proximal emboli located within the right ventricle, main pulmonary artery, or extrapulmonary branches. Notably, patients with isolated distal pulmonary emboli are generally not considered candidates for surgical embolectomy [4].

Management strategies for concurrent PE and ACS vary significantly across the literature. Consistent with the approach taken in this case, previous reports have documented successful outcomes using DOACs as primary therapy [5]. Despite the presence of myocardial injury, as evidenced by elevated troponin and EKG changes, this patient remained hemodynamically stable without a requirement for supplemental oxygen. Following a multidisciplinary review and a shared decision-making process regarding the risks and benefits of invasive intervention, a conservative strategy was adopted, predicated on the patient's clinical stability and rapid symptomatic improvement. Conversely, other reports advocate for thromboembolectomy as a viable alternative for patients with similar clinical profiles [6-7]. These divergent approaches underscore the lack of a defined "gold standard" and highlight the necessity of a nuanced, patient-centered approach to PE-induced cardiac strain which may mimic ACS.

Furthermore, the long-term outcomes associated with conservative management in this specific population have yet to be rigorously investigated. In the broader context of acute PE, catheter-directed thrombectomy has demonstrated lower in-hospital mortality rates when compared to systemic thrombolysis or anticoagulation monotherapy [8]. Conversely, DOACs have proven to be both safe and effective for the treatment of intermediate-risk PE [1]. However, there remains a paucity of comparative data evaluating the efficacy of thrombectomy versus DOACs specifically for the management of PE-induced cardiac strain.

This case report further highlights the need for clinical distinction between ACS and PE mimicking ACS. It further elucidates the current lack of guideline-directed therapy for patients presenting with this dual pathology. While existing protocols provide clear frameworks for managing PE and ACS as independent entities, they offer little guidance for their concurrent management. Continued research and the refinement of clinical pathways are essential to optimize patient outcomes and to better weigh the risks and benefits of various therapeutic interventions. Furthermore, longitudinal studies are warranted to evaluate the long-term complications and prognosis associated with different treatment modalities in the setting of combined PE and ACS.

## Conclusions

PEs are a potentially fatal condition that frequently necessitates individualized management. This case highlights an atypical presentation characterized by ST- and T-wave abnormalities secondary to acute PE. While our patient was successfully managed with anticoagulation following a shared decision-making process, therapeutic approaches remain variable. PE should be considered in the differential diagnosis for patients presenting with acute dyspnea and concomitant troponin elevation. 

## References

[1] Konstantinides SV, Meyer G, Becattini C (2020). 2019 ESC Guidelines for the diagnosis and management of acute pulmonary embolism developed in collaboration with the European Respiratory Society (ERS). Eur Heart J.

[2] (2023). Risk factors for blood clots. https://www.cdc.gov/blood-clots/risk-factors/index.html.

[3] Al-Ogaili A, Ayoub A, Diaz Quintero L (2019). Rate and impact of venous thromboembolism in patients with ST-segment elevation myocardial infarction: analysis of the nationwide inpatient sample database 2003-2013. Vasc Med.

[4] Weinberg Weinberg, A. S., & Rali, P. R. (2025 (2026). Acute pulmonary embolism in adults: treatment overview and prognosis. https://www.uptodate.com/contents/acute-pulmonary-embolism-in-adults-treatment-overview-and-prognosis.

[5] Siddiqa A, Haider A, Jog A, Yue B, Krim NR (2020). Pulmonary embolism presenting as ST-elevation myocardial infarction: a diagnostic trap. Am J Case Rep.

[6] Bruss P, Chauhdri AF, Gombash R (2024). Pulmonary emboli mimicking ST-elevation myocardial infarction patterns. Cureus.

[7] Yue XL, Shi XY, Jiang M, Li RJ (2023). Acute pulmonary embolism presenting with electrocardiographic signs and serum biomarkers of ST-segment elevation myocardial infarction: a case report. J Int Med Res.

[8] Al-Khadra Y, Missula V, Al-Bast B (2024). Outcomes of mechanical thrombectomy compared with systemic thrombolysis in pulmonary embolism: a comprehensive evaluation from the national inpatient sample database. J Endovasc Ther.

